# Supplementary material for: Altered Sensory Neuron Development in CMT2D Mice Is Site-Specific and Linked to Increased GlyRS Levels
Source: Front Cell Neurosci. 2020 Aug 11;14:232. doi: 10.3389/fncel.2020.00232 (PMC7431706; doi:10.3389/fncel.2020.00232)
Supplement: Supplementary file 1 [file Data_Sheet_1.docx]

**Supplementary Figures**

**
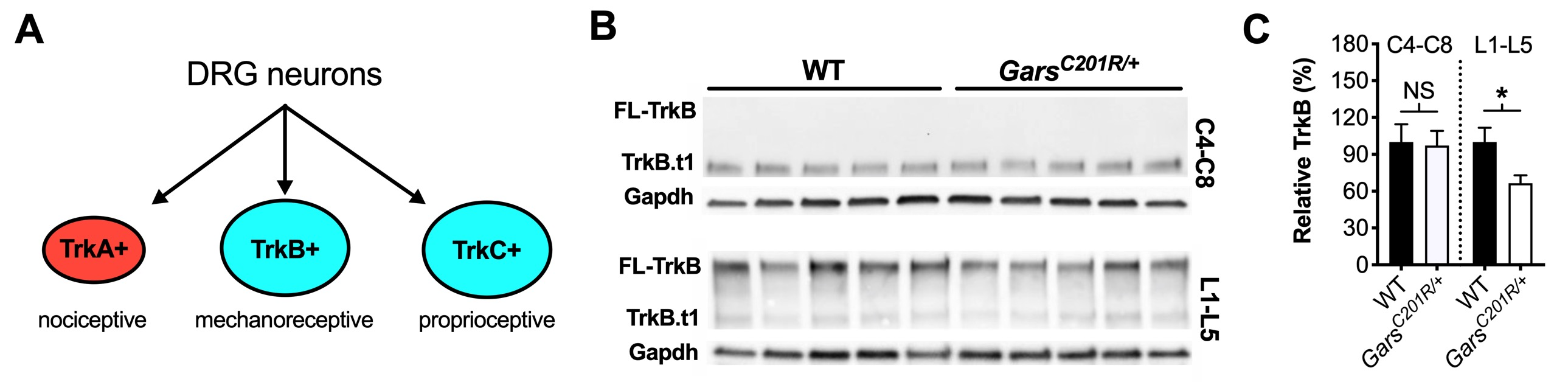
**

**Supplementary Figure 1. TrkB protein levels are lower in *Gars^C201R/+^* lumbar DRG consistent with fewer mechanosensory neurons.** (**A**) Trk receptors A, B and C are expressed in post-natal DRG sensory neurons that function in nociception, mechanosensation and proprioception, respectively (Montaño et al., 2010). TrkA preferentially binds to NGF, TrkB to BDNF and NT-4, and TrkC to NT-3. (**B**) Representative western blot of C4-C8 and L1-L5 DRG lysates from one month old wild-type and *Gars^C201R/+^* mice probed for TrkB and the loading control Gapdh. *N.b.*, samples from the cervical and lumbar levels are not from the same mice, and it is unclear why full-length TrkB (FL-TrkB) is not observed in C4-C8 DRG. *TrkB.t1*, truncated TrkB. (**C**) Densitometry analysis indicates that there was no difference between genotypes in total TrkB levels in cervical ganglia (*P* = 0.882; unpaired *t*-test); however, TrkB was reduced in mutant L1-L5 DRG (* *P* = 0.037; unpaired *t*-test), likely reflecting the previously identified sensory subtype switch in lumbar DRG (Sleigh et al., 2017a). *n* = 5. *NS*, not significant; *WT*, wild-type. Related to **Figure 1**.

**
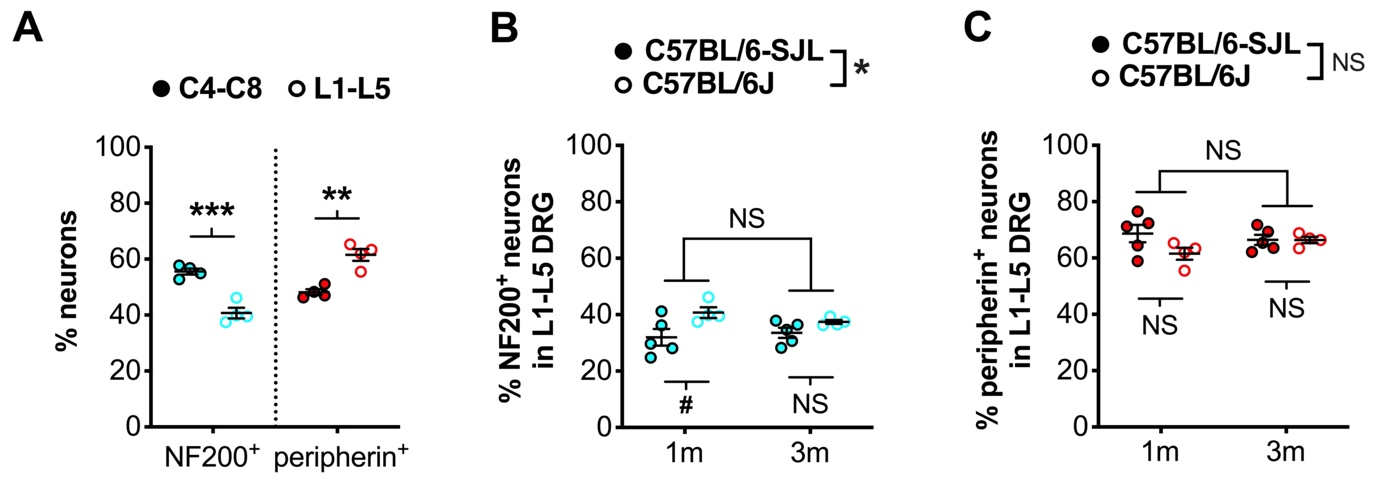
**

**Supplementary Figure 2. A large difference in sensory neuron populations is observed between cervical and lumbar ganglia.** (**A**) C4-C8 DRG possess significantly more NF200^+^ neurons and significantly fewer peripherin^+^ cells than L1-L5 DRG. C4-C8 data are also presented as “*wild-type*” in **Figure 1D**. *** *P* < 0.001, ** *P* < 0.01; unpaired *t*-test. (**B**) There is a small difference in the percentage of NF200^+^ neurons in L1-L5 DRG between mice on a pure C57BL/6J background and a mixed C57BL/6-SJL background (strain * *P* = 0.011, age *P* = 0.694, interaction *P* = 0.277; two-way ANOVA). **^#^** *P* < 0.05, *NS* not significant; Sidak's multiple comparisons test. (**C**) No difference is observed between strains in the percentage of peripherin^+^ neurons in lumbar ganglia (strain *P* = 0.140, age *P* = 0.576, interaction *P* = 0.144; two-way ANOVA). *NS* not significant; Sidak's multiple comparisons test. The one month C57BL/6J data in B and C are presented as “*L1-L5*” in panel A. Data from C57BL/6J lumbar ganglia were generated for a previous study (Sleigh et al., 2017a). C57BL/6-SJL data are also presented as “*wild-type*” in **Figure 3B** and **D**. *N.b.*, statistically compared DRG datasets were independently stained, imaged and quantified, which may account for some variability. *n* = 4-5. *1m*, 1 month; *3m*, 3 months. Related to **Figure 1** and **3**.

**
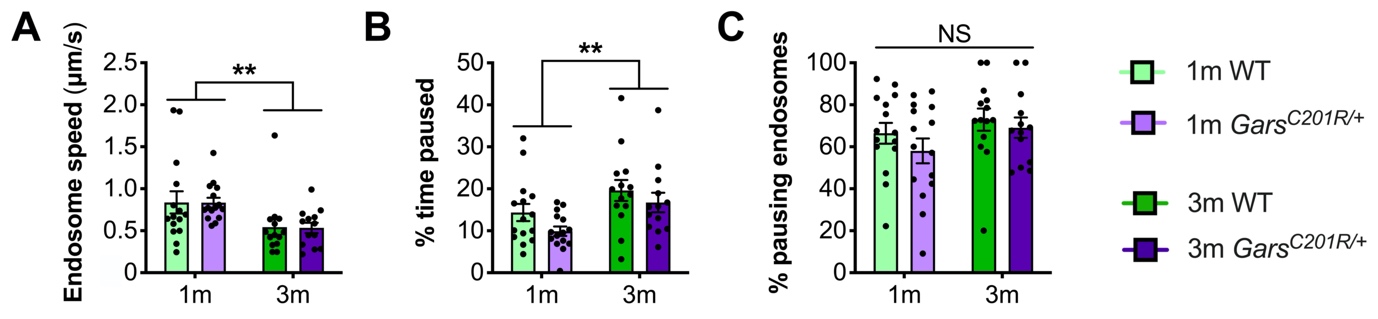
**

**Supplementary Figure 3. No difference in endosome transport between genotypes is found using neuronal process as the experimental unit.** (**A-C**) A lack of distinction in endosome dynamics between genotypes was confirmed using processes as the experimental unit. There is no difference between wild-type and *Gars^C201R/+^* neurons in average endosome speeds (A, genotype *P* = 0.948, age ** *P* = 0.002, interaction *P* = 0.979; two-way ANOVA), percentage of time endosomes paused for (B, genotype *P* = 0.077, age ** *P* = 0.005, interaction *P* = 0.700; two-way ANOVA), or percentage of pausing endosomes (C, genotype *P* = 0.259, age *P* = 0.107, interaction *P* = 0.671; two-way ANOVA). However, there was a significant difference between timepoints in endosome speed (A) and % time paused (B), suggesting that there may be an age-related slow-down in endosome transport in cultured DRG sensory neurons. *n* = 13-15. *1m*, 1 month; *3m*, 3 months; *NS*, not significant; *WT*, wild-type. Related to **Figure 4**.

**
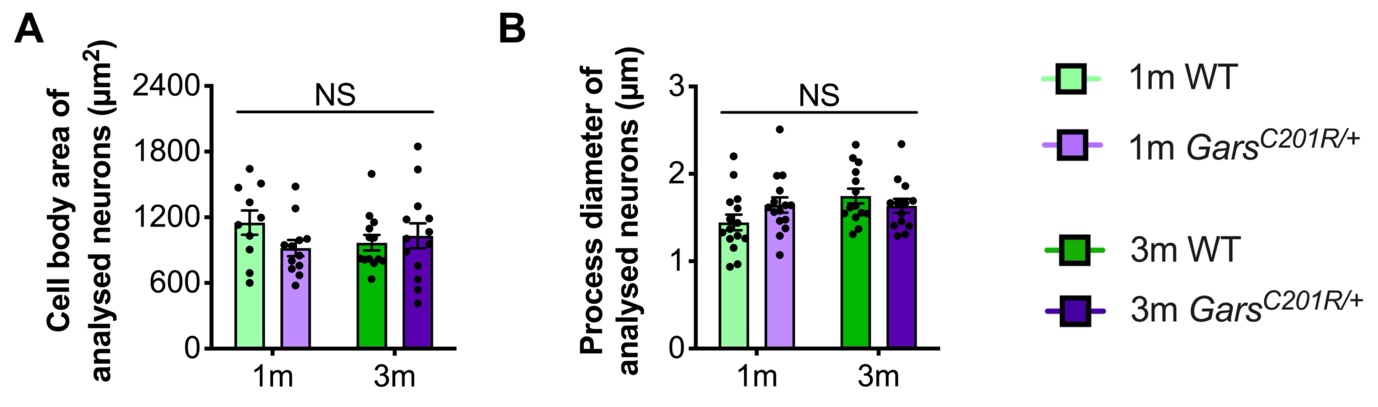
**

**Supplementary Figure 4. Large area sensory neurons of similar size between genotypes were assessed in the endosome transport assay.** To ensure that retrograde transport of signalling endosomes was analysed in similar neuron subtypes between genotypes and across timepoints, the cell body area (**A**) and process diameter (**B**) of each tracked neuron were measured. No difference in either morphological property was detected, indicating that transport was assessed in comparable sensory neuron subtypes. *n* = 4, but with 10-15 individual neurons depicted. Two-way ANOVAs using either animal (*n* = 4) or process (*n* = 10-15) as the test replicate both indicated that there were no significance differences in age, genotype or their interaction. *N.b.,* at the one month timepoint, cell bodies were not measured in one experimental replicate, although their similar size was confirmed visually and their process diameters were no different from all other cultures (B). *1m*, 1 month; *3m*, 3 months; *NS*, not significant; *WT*, wild-type. Related to **Figure 4**.
